# Electronic structure of SrSn_2_As_2_ near the topological critical point

**DOI:** 10.1038/s41598-017-05386-x

**Published:** 2017-07-21

**Authors:** L.-Y. Rong, J.-Z. Ma, S.-M. Nie, Z.-P. Lin, Z.-L. Li, B.-B. Fu, L.-Y. Kong, X.-Z. Zhang, Y.-B. Huang, H.-M. Weng, T. Qian, H. Ding, R.-Z. Tai

**Affiliations:** 1 Shanghai Synchrotron Radiation Facility, Shanghai Institute of Applied Physics, Chinese Academy of Sciences, Shanghai, 201204 China; 2 Beijing National Laboratory for Condensed Matter Physics and Institute of Physics, Chinese Academy of Sciences, Beijing, 100190 China; 3 University of Chinese Academy of Sciences, Beijing, China

## Abstract

Topological materials with exotic quantum properties are promising candidates for quantum spin electronics. Different classes of topological materials, including Weyl semimetal, topological superconductor, topological insulator and Axion insulator, etc., can be connected to each other via quantum phase transition. For example, it is believed that a trivial band insulator can be twisted into topological phase by increasing spin-orbital coupling or changing the parameters of crystal lattice. With the results of LDA calculation and measurement by angle-resolved photoemission spectroscopy (ARPES), we demonstrate in this work that the electronic structure of SrSn_2_As_2_ single crystal has the texture of band inversion near the critical point. The results indicate the possibility of realizing topological quantum phase transition in SrSn_2_As_2_ single crystal and obtaining different exotic quantum states.

## Introduction

**Figure 1:**
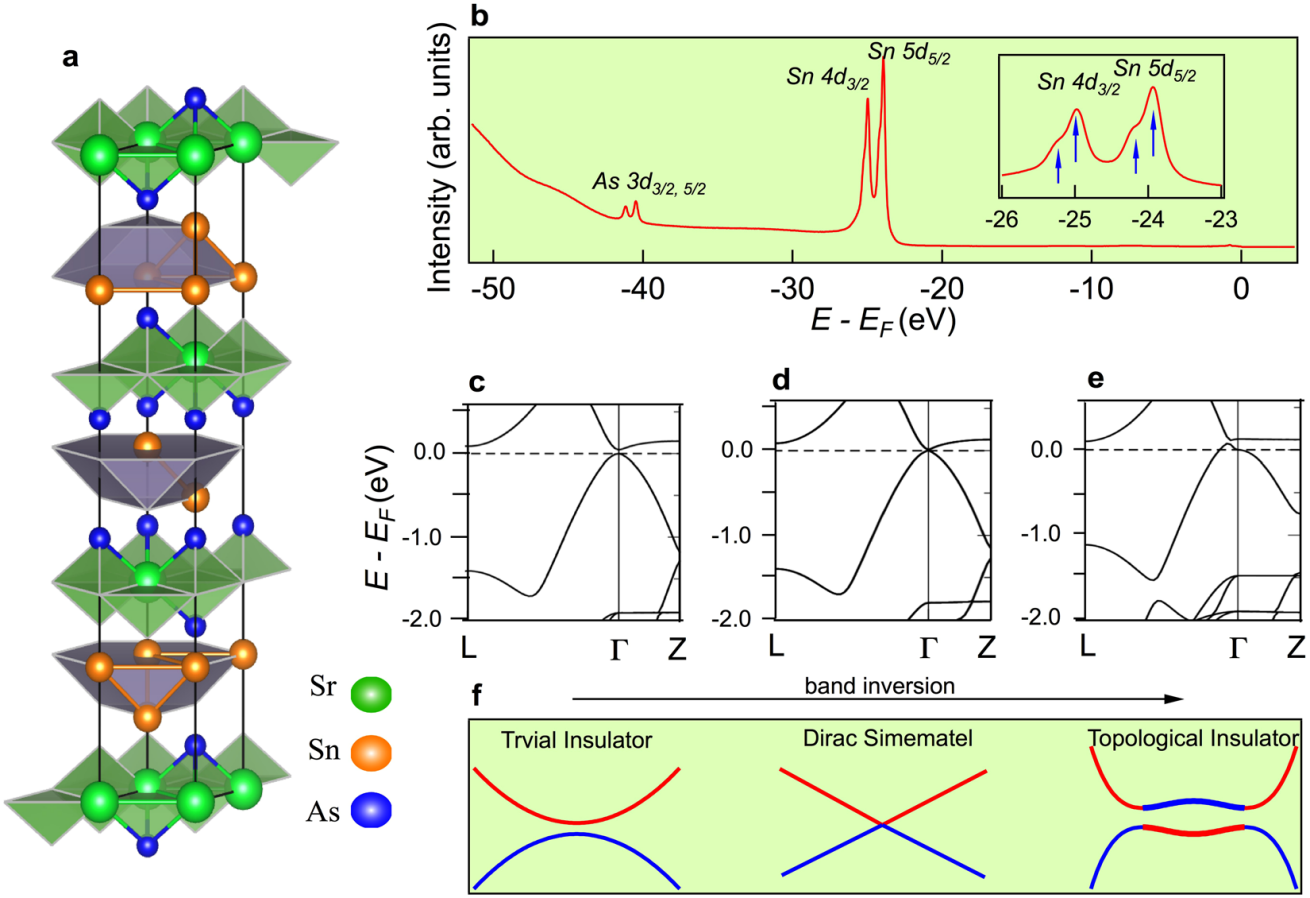
(**a**) Crystal structure of SrSn_2_As_2_. (**b**) Core level photoemission spectrum shows characteristic peaks of As and Sn. (**c**) and (**e**) Calculated bulk band structures with and without mBJ along high symmetry lines shows trivial band insulator and topological insulator state, respectively. (**d**) The critical point between (**c**) and (**e**) shows Dirac semimetal state. (**f**) Schematic of the band inversion mechanism in topological phase transition corresponding to (**c**) to (**e**).

Topological materials attract much attention because of their exotic characters including non-trivial Dirac surface state, magnetic monopole Fermi arc and so on^1–7^. This field has enjoyed rapid development in recent years thanks to the well agreement and effective mutual promotion between experimental observation and theoretical calculation. In recent two years, the experimental discovery of three-dimensional (3D) Dirac semimetal, Weyl semimetal and hourglass fermion follows soon after the theoretical predications^8–12^. Among the families of topological materials, topological insulator is a novel state of matter. It has an energy gap in the bulk like ordinary insulators but gapless Dirac fermionic states on the boundaries. This intriguing feature makes it a potential candidate for spintronic devices due to the special spin properties of the surface state^13^. It is known that trivial band insulator can be tuned into topological insulator through a topological quantum phase transition by modulating the spin-orbital coupling or lattice parameters^14^. Band inversion is the key process in the transition, during which the energy gap between the valence bands and conduction bands becomes smaller and smaller until closed as shown in Fig. 1(f). At this critical point the system turns into the well-known Dirac semimetal. Because of spin-orbit interaction the energy gap will reopen along with the non-trivial Dirac surface states appearing inside it, and the system becomes topological insulator^15–18^. Thus the investigation of materials near the topological critical point will not only help us understand the nature of topological quantum phase transition but also provide new ideas for finding Dirac semimetal materials.

Till now several materials have been predicted to be near the critical point, such as CdTe/GdTe quantum well with specific thickness^15^, ZrTe_5_^19–22^, Ca_2_PtO_4_^23^, Au_2_Pb^24, 25^, Bi_4_X_4_^26, 27^, Bi_2_Te_2_Se^28^ and so on. Most of them have been studied intensively. SrSn_2_As_2_ has been predicted to be a candidate for new 3D Dirac semimetal because it is naturally near the critical point. This prediction is based on the general design principles for predicting 3D Dirac semimetals from charge-balanced semiconductors with symmetry considerations^28^. The question now is whether the real SrSn_2_As_2_ single crystal is normal insulator, Dirac semimetal or topological insulator, and what factors can drive the phase transition. With these questions we systematically investigate the electronic states of SrSn_2_As_2_ single crystal. The ARPES results show the feature of band inversion around Γ point, which is evidence of strong topological insulator state. The experimental results are successfully reproduced by our LDA calculation. By modifying the exchange potential, the ordinary insulator state is also realized in the calculation. Our study shows that the single crystal SrSn_2_As_2_ is probably a new topological insulator near the topological critical point.

## Results and Discussion

### Crystal structure and *ab-inito* calculations

SrSn_2_As_2_ crystals can be regarded as a superlattice consisting of alternating stacking of SrAs_2_ and Sn layers characterized by space group R-3m h (166) as shown in Fig. 1(a). The cleavage mostly takes place between the two layers, leaving the Sn atoms or As atoms outside. We display the core level spectrum of SrSn_2_As_2_ recorded on the *in-situ* cleaved sample. On the enlarged spectrum shown in the inset of Fig. 1(b), there are two extra shoulders near the Sn *4d* and *5d* peaks, meaning that the Sn atoms are on the cleaved surface.

In order to understand the topological band structure of SrSn_2_As_2_, we performed *ab-inito* calculations within the framework of density function theory (DFT) using the VASP^29, 30^. Spin-orbit coupling is taken into account self-consistently. We display the band structure along high symmetry line around Γ in Fig. 1(e). The feature of band inversion can be clearly seen around the Brillouin Zone (BZ) center, contributed by Sn *p*_*z*_ orbit and As *p*_*z*_ orbit. The band inversion results in a nontrivial topological *Z*_*2*_ index of *ν*_*0*_ = 1 according to the *Z*_*2*_ classification^31^. This result indicates that the single crystal of SrSn_2_As_2_ is a strong topological insulator with band inversion similar to the Bi_2_Se_3_ family^1, 32^. But when we redo the calculation with the modified Becke-Johnson exchange potential (mBJ)^33^, the topological state is different as shown in Fig. 1(c), we get a trivial insulator with no band inversion, which means that this crystal can theoretically be tuned from a strong topological insulator into a trivial insulator. Between the two phases we can get a Dirac semimetal state Fig. 1(d). Our calculation suggests that the single crystal SrSn_2_As_2_ is near the topological critical point.

### Electronic structure of SrSn_2_As_2_

**Figure 2:**
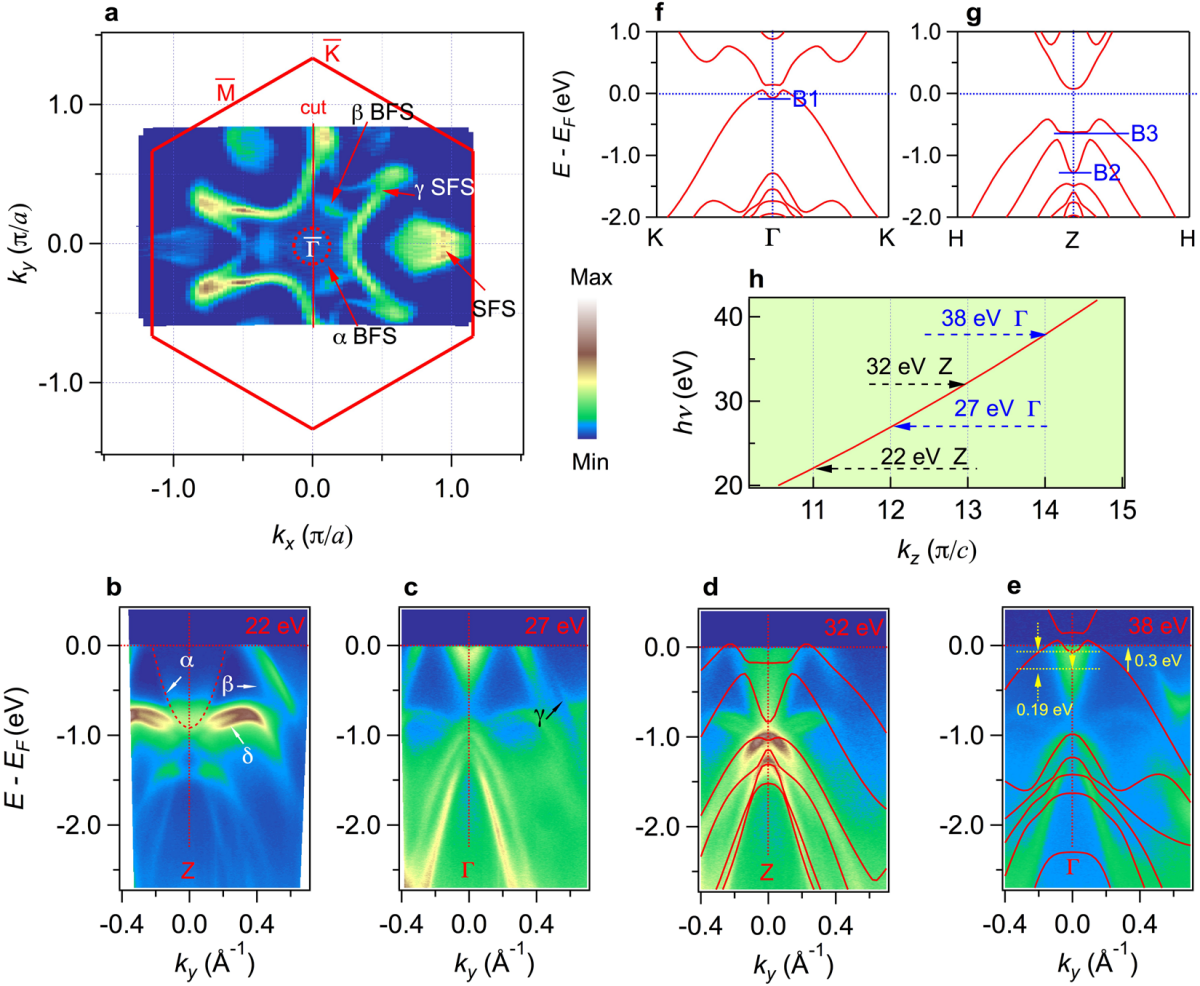
Fermi surfaces and photon energy dependence of band dispersion of SrSn_2_As_2_. (**a**) Fermi surface intensity plot of single crystal SrSn_2_As_2_ recorded at *hv* = 22 eV and T = 25 K with linear horizontal polarization. (**b**) to (**e**) Bands dispersion along cut recorded with different photon energy, respectively. LDA calculated bands are plotted on the experimental data for comparison. (**f**) and (**g**) Calculated bulk band structure along high symmetry line at *k*_*z*_ = 0 and *k*_*z*_ = π/*c*, respectively. (**h**) The fitted photon energy curve versus *k*_*z*_.

To study the electronic structure and topological character of SrSn_2_As_2_, we performed systematic ARPES measurement on the 001 surface. Figure 2(a) shows the Fermi surface intensity map at *hv* = 22 eV and *T* = 25 K. We can see the 3-fold Fermi surfaces around Γ and dumb-bell like Fermi surfaces at BZ boundary. The band structure along high symmetry line labeled as “cut” with red line in Fig. 2(a) is displayed in Fig. 2(b–e). We identify three Γ-centered bands crossing *E*_*F*_, the inner one marked as α is an electron band, the outer ones marked as β and γ are hole-like bands. α and β band contribute to two small Fermi surface pockets around BZ center marked as α BFS (bulk Fermi surface) and β BFS in Fig. 2(a) while γ forms the big flower like Fermi surface marked as γ SFS (surface Fermi surface). There are also two bands nearly parallel to each other marked as δ bands located at about −0.8 eV. When we change the photon energy from 20 eV to 40 eV, the bottom of α electron band moves from −1 eV to −0.2 eV periodically, the Fermi vector of α band changes between 0.21 Å^−1^ and 0.06 Å^−1^, and the Fermi vector of β band also changes between 0.35 Å^−1^ and 0.30 Å^−1^ periodically. The γ band remains the same within our resolution. According to the variation period of α and β bands along photon energy, we can fit the *hv* versus *k*_*z*_ curve very well in Fig. 2(h) using inner potential *V*_*o*_ = 12.2 eV and lattice parameter *c′* = 26.728/2 Å. It should be noted that the *c*’ used here is half of the lattice constant *c* because there are two equivalent layers in one unit-cell along *z* direction as shown in Fig. 1(a). The difference in their photon energy dependence indicates that α and β bands are bulk states while γ band is surface state. Figure 2(f and g) show calculated band structure along the high symmetry line in the plane of *k*_*z*_ = 0 and *k*_*z*_ = π/*c* respectively. The band structure contains only one ‘M′ shape valance band near Fermi level around Γ. On *k*_*z*_ = 0 plane, the bottom of the valance band at Γ point (marked as B1) is around 0.1 eV below *E*_*F*_. while on *k*_*z*_ = π/*c* plane, the bottom (marked as B2) shifts down to around 1.2 eV below *E*_*F*_ (the other bottom above marked as B3 results from hybridization of conduction band and the neighbor band below it). In order to reproduce the experimental data, in the *k*_*z*_ = 0 plane we shift the LDA calculated valence bands up about 0.3 eV, and the calculated conductance bands down about 0.19 eV, the agreement between them is much better than before in Fig. 2(e). In the *k*_*z*_ = π plane, all the bands below *E*_*F*_ are valence bands, when they are shifted up about 0.45 eV, the consistence becomes very well. The different shifting directions of valence bands and conductance bands indicate that the main cause of the discrepancy between the calculation and the experiment is the underestimation of the inversion size between the valence and conduction bands in calculations, which is a common shortcoming of LDA or GGA calculations in semiconductors. Considering the underestimation of the band gap by GGA, we have checked the band inversion carefully. The band topology of the occupied states is invariant even if the band inversion is underestimated. Further calculation information shows that α band is an inverted band contributed by Sn *p*_*z*_ orbit and β band is contributed by As *p*_z_ orbit.

**Figure 3:**
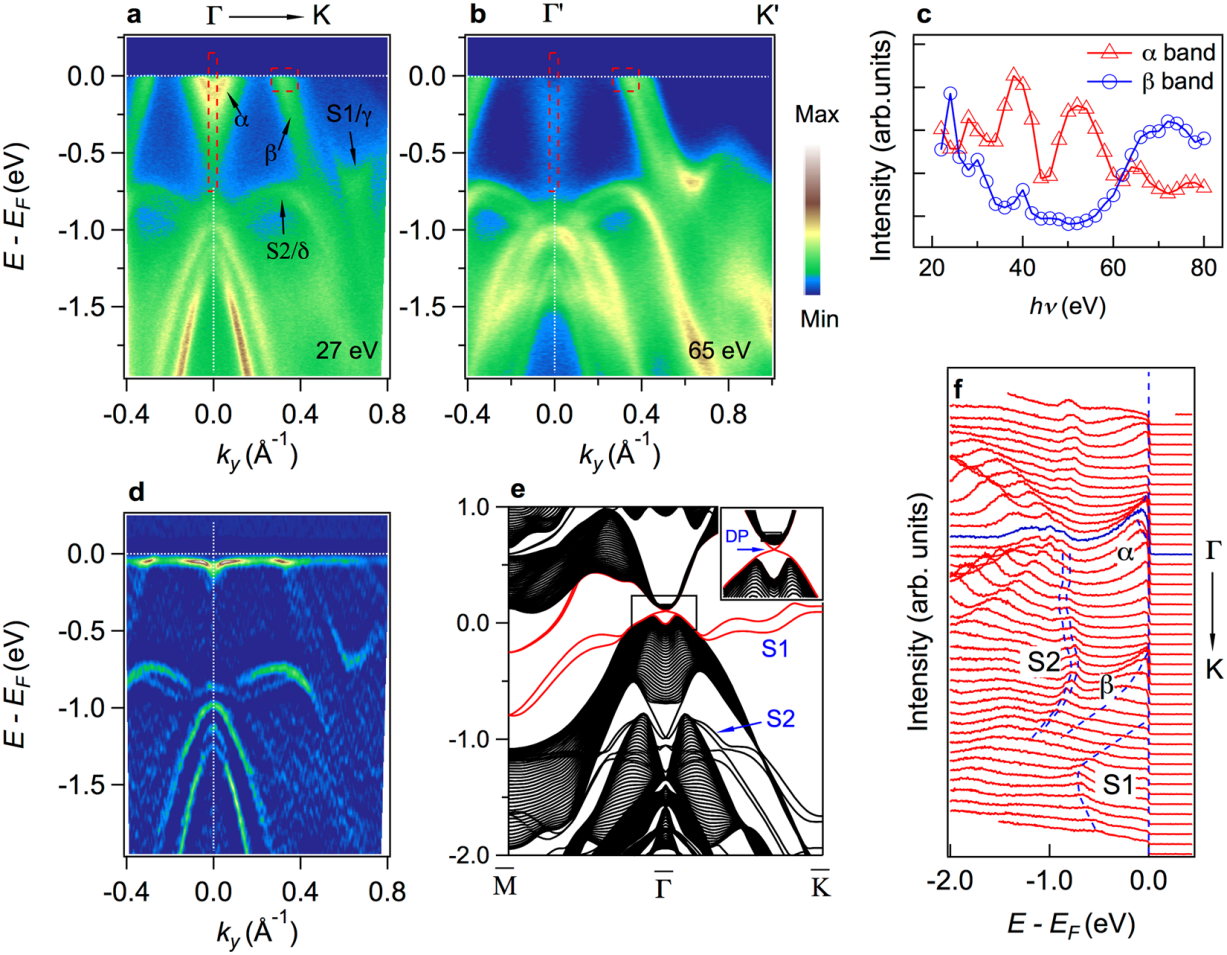
Texture of band inversion and surface states compared with calculations. (**a**) ARPES intensity plot of band structure along Γ-K recorded with *hv* = 27 eV. (**b**) Same as (**a**) but *hv* = 65 eV. (**c**) The integrated intensity of α band and β band versus photon energy. (**d**) Curvature intensity plot of (**a**). (**e**) The calculated surface states of SrSn_2_As_2_ (001) surface. The inset shows the Dirac Point (DP). (**f**) Energy distribution curves (EDCs) of (**a**).

After discussing the *k*_*z*_ dependent data, now let’s focus on the surface states along K-Γ-K. In order to identify the surface states, we display the calculated surface states of SrSn_2_As_2_ (001) surface in Fig. 3(e). We get the surface Dirac cone in the bulk band gap at Γ point as shown in the inset of Fig. 3(e). However, the Dirac point in experiments is above the Fermi level because the chemical potential is lower than the calculation, and our ARPES cannot reach the unoccupied electronic states. In the calculation result shown in Fig. 3(e), the lower branches of the cone marked as S1 extend to the energy gap on the BZ boundary rather than merge into the bulk bands. These bands are the γ band observed in Fig. 2. The two nearly parallel δ bands in our experiment spectrum are also reproduced in the calculation and marked as S2 in Fig. 3(e). They are also surface states but not so important to the topological character. The details of the surface bands do not agree well with the calculations. In topological insulators, the nontrivial topology is usually defined by topological invariant of the bulk bands. If the Berry phase is non-zero nontrivial, the topological boundary states will always exist. The details of the surface states depend on the boundary, for example, different cleavage termination, surface reconstruction, surface band bending effect and so on. Therefore, if the bulk bands hold nontrivial band inversion, the Dirac surface states will be there no matter what the details look like.

The greatest contribution to the texture of band inversion near *E*_*F*_ comes from α band and β band. From Fig. 3(a), it is clearly shown that the bottom of electron band (conductance band/α band) is lower than the top of the hole band (valence band/β band). This cannot happen in the calculated trivial insulator states as we have shown in Fig. 1(c–e) and the schematic Fig. 1(f), which means the appearing of α band below the top of β band can only happen in nontrivial band inversed topological insulator state. To distinguish different orbital components induced by band inversion, we display in Fig. 3(c) the intensity of integrated photoelectron spectrum of α band and β band versus photon energy respectively, the two different dotted rectangle boxes in Fig. 3(a and b) indicate the range of integration of α band and β band. It is known that the photoelectron intensity of a specific band is positively related to the photoionization cross-sections, asymmetry parameters and matrix element effect^34, 35^. For bands consisting of different orbital components, the curves of photoelectron intensity versus photon energy usually have different shapes. We can see in Fig. 3(c) that when photon energy is changed from 20 eV to 80 eV, the red curve is generally decreasing except for some large fluctuations. Above 60 eV the intensity of α band becomes too weak for us to track the band dispersion, as shown Fig. 3(b). For the blue curve, the intensity reaches the minimum at about 50 eV before rising again. It is worth noting that the peaks at about 20 eV and 40 eV are formed from inner shell electrons excited by the second harmonic component in the synchrotron radiation, whose photon energy is near the core-level shown in Fig. 1(b). The difference between the shapes of the two curves indicates that α band and β band come from different orbital components, the α band comes from the inverted conductance band while the β band comes from the valence band. From what has been discussed above, the experiment results show the evidence of band crossing between conductance band and valence band in SrSn_2_As_2_. In our calculation, if the crossing happens, the gap will be open considering SOC, resulting in nontrivial Berry phase around the inverted Fermi surface pockets. Thus, we can draw the conclusion that the α band is evidence of band inversion texture.

## Conclusion

In summary, we investigated the electronic structure of SrSn_2_As_2_ and observed the texture of band inversion and topological surface states. Our experiment results are reproduced by the calculation. The calculation suggests that single crystal SrSn_2_As_2_ is near the topological critical point between trivial insulator and a strong topological insulator. In the calculation using modified Becke-Johnson exchange potential the trivial insulator state appears. The direct evidence of band inversion from ARPES data indicates the probable strong topological insulator state of this crystal. Our studies provide the evidence for a new topological material near topological critical point, which is a potential material for further studies on topological quantum phase transition in the future.

## Methods

Single crystals of SrSn_2_As_2_ were grown by the solid-state reactions. Stoichiometric amounts of Sr (lump, 99.7%), Sn (shot, 99.999%%) and As (lump, 99.99%%) were sealed in an sealed quartz ampoule backfilled with about 0.2 atm high pure argon and placed in a furnace. Typical temperature gradient from 850 °C to 650 °C was applied. After seven days, bulk single crystals were obtained. The elemental compositions were checked by Oxford X-max energy dispersive X-ray spectroscopy (EDX) analysis in a Hitachi S-4800 scanning electron microscope. The atom percentage ratio between Sr, Sn and As is 1:2:2 with the error bar less than 1%, which is within the systematic error. We also conducted Hall conductivity measurement to check the carrier concentration, and the result indicates that the sample is not considerably hole-doped.

ARPES measurements were performed at the “Dreamline” beamline of the Shanghai Synchrotron Radiation Facility (SSRF) with a Scienta D80 analyzer. The energy and angular resolutions were set to 15–30 meV and 0.2°, respectively. The samples for ARPES measurements were cleaved *in situ* and measured in a temperature range between 20 and 50 K in a vacuum better than 5 × 10^−11 ^Torr.

The Vienna *ab initio* simulation package (VASP)^29, 30^ is employed for first-principles calculations. The generalized gradient approximation (GGA) of Perdew-Burke-Ernzerhof type^36^ is used for the exchange-correlation potential. Spin-orbit coupling (SOC) is taken into account self-consistently. The *k*-point sampling grids for different structures have been tested to be dense enough. The atomic structure and the lattice constants *a* = *b* = 4.204 Å, and *c* = 26.728 Å are adapted in our calculations^37^.
